# Survivability of freeze- and spray-dried probiotics and their effects on the growth and health performance of broilers

**DOI:** 10.14202/vetworld.2023.1849-1865

**Published:** 2023-09-17

**Authors:** Juthamas Buahom, Surasak Siripornadulsil, Peerapol Sukon, Treerat Sooksawat, Wilailak Siripornadulsil

**Affiliations:** 1 Department of Microbiology, Faculty of Science, Khon Kaen University, Khon Kaen 40002 Thailand; 2 Research Center for Environmental and Hazardous Substance Management, Khon Kaen University, Khon Kaen 40002, Thailand; 3 Faculty of Veterinary Medicine, Khon Kaen University, Khon Kaen 40002, Thailand

**Keywords:** broilers, growth performance, immobilized probiotics, intestinal morphology, lactic acid bacteria

## Abstract

**Background and Aim:**

Many strains of probiotics have been exploited and used as animal dietary supplements for broiler production. The efficacy and survival of probiotics during production may reflect better activities of the probiotics in the host. This study investigated the effects of freeze- and spray-drying on the survivability and properties of probiotics and their ability to improve the growth and health performance of broilers.

**Materials and Methods:**

Probiotic powders of four strains of lactic acid bacteria, *Enterococcus faecium* CA4, *Enterococcus durans* CH33, *Ligilactobacillus* *salivarius* CH24, *Pediococcus acidilactici* SH8, and *Bacillus subtilis* KKU213, were prepared using rice bran/chitosan/carboxy methyl cellulose as the carrier. The survival of each probiotic strain was investigated under stress conditions, including freeze-drying, spray-drying, and simulated gastrointestinal conditions. The body weight gain (BWG) and intestinal histomorphology were determined to assess broiler growth performance.

**Results:**

All dried probiotics yielded a high survival rate during freeze-drying (95.8–98.6%) and spray-drying (94.4–98.2%). In addition, an analysis of the main effect revealed that the effectiveness of freeze-drying was higher than that of spray-drying in minimizing the loss of cell viability. The antimicrobial activity of all immobilized dried probiotic strains against *Salmonella* was maintained. The immobilized probiotics tolerated a low pH value of 2.0 and 0.5% (w/v) bile salt. Probiotic administration of a mixture of the five dried probiotics to 1-day-old hatched male broilers at early and late ages resulted in potential colonization in the broiler intestine, and enhancements in the BWG, lipid metabolism, and gut health (villus height and cryptal depth) were observed in the probiotic-treated groups.

**Conclusion:**

The administration of three doses of the spray-dried probiotic mixture at days 15, 17, and 19 after hatching was sufficient to achieve long-term growth and health benefits in broilers. This finding might provide a cost-effective alternative to the administration of commonly used antibiotics in broiler production.

## Introduction

The demand for broiler meat products has increased rapidly worldwide. Broilers are bred and raised to achieve optimal body weight within 35–45 days. However, the growth and health of broilers depend on the strain, feeding, and rearing conditions, which must be considered to achieve maximum productivity [1]. Nutrition and dietary supplementation are important for enhancing growth performance and preventing disease events in broiler farms. The administration of dietary antibiotics is practical in poultry husbandry [2]. The inappropriate use of antibiotics as growth stimulants and therapeutic agents in animal farms triggers increases in antibiotic resistance and risks associated with the deposition of antibiotic residues in animal products [3], increases in the emergence of multidrug-resistant bacterial strains, and disruption of the normal commensal microbiota [4]. Moreover, poor practices related to the long-term use of antibiotics have contributed to the distribution of multidrug-resistant pathogens in the environment surrounding animal farms and meat products [4]. Therefore, an alternative method involving feed supplementation with additives, particularly probiotics, has been suggested to increase the growth performance of food animals and protect against pathogen infection [3]. Probiotics are living microorganisms that have beneficial health impacts on the host when administered in adequate amounts (Food and Agriculture Organization). Most bacterial probiotics are classified as *Bacillus*, *Bifidobacteria*, *Enterococcus*, *Lactobacillus*, *Lactococcus*, *Leuconostoc*, *Pediococcus*, and *Streptococcus* and provide advantages for animal health through inhibitory activities against pathogens, modulation of immunity and hematology in the animal host, and improvements in the intestinal barrier [5]. In addition, the microbiological quality and sensory characteristics of broiler meat could be improved by probiotic administration. The oral administration of probiotics through feed and water supplementation is a commonly used method in animal husbandry.

Immobilization and microencapsulation have been suggested as adequate technologies for protecting probiotic bacteria from the detrimental conditions to which bacterial cells can be exposed during drying [6]. Carrier agents such as maltodextrin, gum arabic, calcium-alginate, inulin, and chitosan have been commonly used for immobilization purposes, and their use has helped maintain the viability and stability of probiotics under storage conditions and in the host’s gastrointestinal tract [7]. Based on their properties, some polysaccharides and oligosaccharide carriers can be regarded as potential prebiotics that cannot be hydrolyzed and absorbed by the host’s gastrointestinal tract. Prebiotics can selectively stimulate numerous probiotics and a limited number of bacteria in the intestinal tract [8]. The material extracted from agricultural wastes comprising cellulose, hemicellulose, and lignin has been cost-effectively used for many beneficial purposes. The advantages of soluble fiber extracted from soybean solid waste, oil palm trunk, and oil palm frond for probiotic immobilization have been demonstrated [9]. The co-immobilization of probiotic bacterial cells with one or more different types of carrier material, such as rice bran, chitosan, and other cellobioses, results in a high number of viable cells after exposure to simulated gastric juice (SGJ) and simulated intestinal juice (SIJ) [10]. However, some limitations in large-scale production are the specific protectants or carriers for bacterial species that can reduce the harmful effects observed after encountering high processing temperatures. Therefore, the large-scale preparation of viable probiotics for industrial use is challenged by their low survival rate during preparation by carriers with insufficient proportions or inappropriate types [6]. The inefficacy of probiotic production can lower the survival rate of thermally inactivated probiotics, which impacts the number of surviving probiotics in the host intestine. The high survival of probiotics in the host intestine may reflect better activities of the probiotics in the host. Dried yeast extract, chitosan, and carboxymethylcellulose (CMC) have been used as carriers and have shown effectiveness as prebiotics to deliver probiotics and improve the egg production and quality and the immune response of laying hens [11]. According to our results, laying hens were less motivated to consume feed-containing carrier with the strong smell of dried brewer’s yeast (unpublished data). Hence, rice bran was used instead of dried brewer’s yeast as a prebiotic-carrier in broilers.

This study aimed to evaluate the survivability of dried probiotics immobilized on a mixture of prebiotic carriers (rice bran, chitosan, and CMC) and their efficacy on broiler performance when added to the diet. The survival of each probiotic strain under stress conditions, including freeze-drying, spray-drying, and simulated gastrointestinal conditions, was investigated. To evaluate the potential application of a mixture of prebiotics and probiotics in broiler production, the growth performance, health status, and intestinal morphology of probiotic-fed broilers were monitored.

## Materials and Methods

### Ethical approval

All animal experiments were conducted in accordance with the guidelines and recommendations of the Institutional Animal Care and Use Committee and approved by the Animal Care Committee at Khon Kaen University, Khon Kaen, Thailand (Approval No. AEKKU 22/2558).

### Study period and location

The study was conducted in April to May 2015 at the Kuru Aood Farm in Sakon Nakhon, Thailand, where broilers were raised in semi-open housing [12]. Each group of broilers was raised separately in thick-black plastic nests on the floor. During the experiments, continuous light was provided throughout the experiment, and the house temperature was approximately 30-35°C.

### Chemicals, reagents, and microbiological media

All chemicals and reagents used for all analytical procedures were of analytical grade with a purity higher than 99.0% and were purchased from Sigma-Aldrich (USA), VWR (UK), Carlo (Spain), and Merck (Germany). Microbiological media, namely, Luria-Bertani (LB, USA), de Man, Rogosa and Sharpe (MRS), and xylose lysine deoxycholate (XLD), were purchased from Difco and BBL (USA).

### Bacterial strains

The probiotic strains used in this study have been deposited in the NCBI database: *Bacillus subtilis* KKU213 (KF220378) and four strains of lactic acid bacteria (LAB), namely, *Enterococcus faecium* CA4 (MF066894), *Enterococcus durans* CH33 (MF066896), *Ligilactobacillus* *salivarius* CH24 (MF066895), and *Pediococcus acidilactici* SH8 (MF061302) [11, 12]. Working stock cultures of each probiotic for freeze-drying were prepared by inoculating a single colony into 10 mL of MSM (for LAB probiotics) or LB (for *B. subtilis*) and incubating at 37°C for 48 h or 18 h, respectively. Working stock cultures for spray-drying were prepared by culturing a single colony of each probiotic in 10 mL of modified broth (1% w/v peptone, 0.3% w/v beef extract and 2% glucose) at 37°C, and the incubation periods for LAB and *B. subtilis* were 48 h and 18 h, respectively. The working stock cultures were used within 1 h of preparation.

### Prebiotic carriers

Three types of commercial oligosaccharide carriers, regarded as prebiotics, rice bran, chitosan, and CMC, were purchased from local companies. All prebiotics were sterilized by heating using an autoclave at 121°C and 15 psi for 15 min. Sterile rice bran, chitosan, and CMC were mixed in a ratio of 6:1:1 (w/w/w) using aseptic techniques to prepare a sterile mixture of prebiotic carriers**.**

### Cell immobilization by freeze-drying and spray-drying

#### Freeze-drying

Starter cultures of LAB probiotics were prepared by adding 2% (v/v) working stock cultures to 100 mL of MRS broth and incubating at 37°C for 48 h, and the starter culture of *B. subtilis* KKU213 was prepared in 100 mL of LB broth and incubated at 37°C for 18 h. Afterward, 20 mL of each starter culture was added to 10 g of the sterile mixture of prebiotic carriers. The combination of the probiotics and prebiotic carriers was incubated at 37°C and shaken at 150 rpm for 30 min, frozen at −20°C for 24 h, and immediately transferred to a freeze dryer (LaboGene™, Lemvig, Denmark) for 72 h of freeze-drying. The freeze-drying conditions were set to 20°C–50°C, over 0.5 h at 0.1000 mbar. The freeze-dried probiotics were maintained at 4°C in a foil zip-lock bag for 9 months. The survivability of freeze-dried probiotic bacteria during storage was determined every 30 days using the 10-fold dilution and spread plate technique. The freeze-drying of sample was performed in triplicate.

#### Spray drying

The 10% (v/v) working stock cultures previously prepared in modified broth were added to 100 mL of modified broth containing 30 g of the sterile prebiotic-carrier mixture. The probiotics LAB and *B. subtilis* KKU213 incorporated in modified broth with the prebiotic-carrier mixture were then grown at 37°C and shaken at 150 rpm for 48 h and 18 h, respectively. After the incubation period, 100 mL of cell culture broth containing the sterile prebiotic-carrier mixture was dried using a Mini Spray Dryer B–290 (BÜCHI Labortechnik AG, Flawil, Switzerland) with a feed flow rate of 70 mL/h and inlet and outlet temperatures of 120°C and 70°C, respectively. The dried probiotics were stored in a desiccator at room temperature for 6 months, and the viable cell count was evaluated every 30 days using the 10-fold dilution and spread plate technique. The spray-drying of sample was performed in triplicate.

### Survival rate and specific rate of degradation

To determine the viable cell count of each probiotic before freeze- and spray-drying, 1 mL of the mixture of the probiotic and prebiotic-carrier mixture was collected for the enumeration of bacteria by 10-fold dilution in 0.85% NaCl, and an aliquot (0.1 mL) of each dilution was spread onto MRS agar for LAB and onto LB agar for *B. subtilis* KKU213 and incubated at 37°C for 48 and 18 h, respectively. At each sampling time, a small amount (0.1 g) of the freeze- and spray-dried probiotics was diluted 10-fold serially with 0.9 mL of 0.85% NaCl. The suspension was homogenized thoroughly using a vortex mixer, and 0.1 mL of each dilution was spread onto medium plates to enumerate LAB and *B. subtilis* KKU213 as described above. All bacterial colonies grown on the media were counted and calculated as log colony forming units (CFU)/g carrier-probiotic mixture. The enumeration of probiotic numbers was performed in triplicate.

The percent survival rate of the bacteria remaining in the dried powder was calculated as follows: survival rate (%) = N/N_0_ × 100, where N refers to the viable cell count after the drying process or after a particular storage time and N_0_ is the viable cell count before the drying process or at the beginning of storage. The specific rate of degradation (k, day^−1^) of the dried probiotics was calculated as a first-order reaction with k = log(N/N_0_)/t, where N_0_ and N represent the viable cell counts at the beginning of storage and after a particular storage time, respectively, and t denotes the storage time [13].

### Antimicrobial activity of immobilized probiotics

The antimicrobial activity against the pathogenic bacteria *Salmonella* *enterica* serovars Typhimurium, Braenderup, and Enteritidis was determined using agar well diffusion and a double layer method. The immobilized probiotics (0.01 g) obtained by freeze- or spray-drying were placed in 6-mm-diameter wells of an MRS plate and then covered with 50 μL of MRS agar. Luria***-***Bertani agar (7 mL) was poured over the surface of the MRS plate, and the plate was incubated until the LB agar solidified. Subsequently, 10^7^ CFU/mL of each *Salmonella* serovar was swabbed onto the surface agar and incubated at 37°C for 24 h. The inhibition zones were measured to investigate the antimicrobial activity of the immobilized probiotics. The experiment was performed in triplicate.

### Survivability of the immobilized probiotics in simulated gastrointestinal conditions

The freeze- and spray-dried probiotics (0.1 g) were transferred into a microcentrifuge tube containing 0.9 mL of sterile SGJ (phosphate buffered saline [PBS], pH 2.0, containing 125 mM NaCl, 7 mM KCl, 45 nM NaHCO_3_, and 3 g/L pepsin), incubated at 45°C and shaken at 150 rpm for 2 h. The sterile SGJ was prepared and filtered through a 0.2-μm membrane filter. After 2 h of incubation in SGJ, the samples were subjected to two cycles of centrifugation at 4°C and 3047× *g* for 2 min and washed with 0.85% (w/v) normal saline solution before subsequent exposure to SIJ conditions. The bacterial pellets were subsequently resuspended in 9 mL of sterile SIJ (PBS buffer, pH 7.0, containing 0.5% (w/v) bile salt and 2 mg/mL trypsin), incubated at 45°C and shaken at 150 rpm for 3 h. The survival ability of the immobilized bacteria after exposure to the SGJ and SIJ environments for 1, 2, and 3 h was determined. The experiments investigating the survival of the probiotics in both SGJ and SIJ were performed in triplicate.

The number of each LAB probiotic and *B. subtilis* KKU213 remaining at each sampling time under both SGJ and SIJ conditions was enumerated using the spread plate technique in triplicate. An aliquot (0.1 mL) of the samples was spread on the MRS and LB agar plates for the LAB strains and *B. subtilis* KKU213, respectively [12]. The survival of the bacterial probiotics after exposure to SGJ and SIJ conditions was calculated and expressed as the percent survival rate.

### Effects of immobilized probiotics and administration program in male Cobb broilers

One-day-old healthy male Cobb broilers (n = 105) were provided by a commercial company and randomly allocated to seven groups (three chicks per replicate and five replicates per group). Two groups were fed the control diets: Negative control (NC; unheated diets) and positive control (PC; preheated diets). In addition, five groups were fed three different supplemental additives at different stages based on the broiler age (different administration programs).

A commercial basal diet containing undefined antibiotics and anticoccidial drugs served as a control diet for the PC group, and a commercial basal diet without antibiotics and anticoccidial drugs served as a control diet for the NC group. The supplemental additives used in the experiment included (i) prebiotic mixture (PreB), (ii) 1 × 10^8^ CFU/g freeze-dried probiotic mixture (FP_5_), and (iii) 1 × 10^8^ CFU/g spray-dried probiotic mixture (SP_5_). PreB contained sterile rice bran, chitosan, and CMC at a ratio of 6:1:1: The FP_5_ was prepared by mixing five freeze-dried bacterial strains (*B. subtilis* KKU213, *E. faecium* CA4, *E. durans* CH33, *L. salivarius* CH24, and *P. acidilactici* SH8) with the prebiotic-carrier mixture at approximately 2 × 10^7^ CFU of each strain per 1 g mixture. The SP_5_ was prepared by mixing the five spray-dried bacterial strains using a process similar to that used for preparation of the FP_5_.

FP_5_ or SP_5_ was administered to broilers using two administration programs: (1) early-stage (1^st^ week, at 1, 3, and 5 days of age) and (2) late-stage (3^rd^ week, at 15, 17, and 19 days of age). The simple practical procedure used to administer probiotics was performed by placing dried FP_5_ or dried SP_5_ on the feeding trough at the ratio of 15 g probiotics or 1 g probiotics per individual during the early morning hours on the specific days of each administration program. The broilers in the PreB group were fed the prebiotic-carrier mixture daily using a procedure similar to that used to administer probiotics. The broilers in all control and treatment groups were raised using three diets for different specific ages until they reached 45 days of age in semi-open housing. The three formulas of commercial basal diets consisted of corn-soybean meal, 3% fat, 5% fiber, and a 12% mixture of different crude protein contents, including 21% crude protein (starter diet), 19% crude protein (grower diet), and 17% crude protein (finished diet) for broilers aged 1–20, 21–38, and 39–45 days, respectively. The basal diet composition was provided by a commercial manufacturer. Furthermore, the commercial basal diets were heated at 70°C for 48 h to break down or inactivate the antibiotics to at least 50–80% [14] before feeding the animals in the NC group and all groups treated with probiotics and the prebiotic-carrier mixture, except for those in the PC group fed unheated diets.

### Growth performance

The body weight gain (BWG) of the individual animals in each treatment and control group was measured at 1, 7, 14, 21, 35, and 42 days of age.

### Serological analysis

All blood parameters of 45-day-old broilers were determined using BioMajesty® JCA-BM6010/C-DiaSys Diagnostic Systems GmbH (Germany) accordinging to the protocols recommended by the manufacturer. Cholesterol (CHOL) was measured using a CHOL oxidase-peroxidase enzymatic photometric test. Triglyceride (TG) level was measured by a colorimetric enzymatic test using glycerol-3-phosphate-oxidase. A selective enzymatic CHOL measurement was used to measure the high-density lipoprotein (HDL) levels using antibodies targeting human lipoproteins, which can form complexes with low-density lipoproteins (LDL), very LDL, and chylomicrons. A two-step method was used to determine the LDL level. Low-density lipoproteins are protected, whereas non-LDL lipoproteins are enzymatically processed. Afterward, LDL was released, and the level of LDL-CHOL was selectively determined using an enzyme reaction that produced color. N-ethyl-N-(hydroxy-3-sulfopropyl)-m-toluidine (TOOS) was used in an enzymatic photometric test to quantify the level of uric acid. All blood parameters are presented in units of mg/d.

### Enumeration and identification of bacteria in the cecum

One broiler was randomly selected from each replicate of the experimental and control groups and euthanized by cervical dislocation, and the cecum was collected. One gram of cecal sample was rinsed twice with 0.85% NaCl, gently ground using a sterile mortar and diluted 10-fold with 0.85% NaCl. The sample dilutions were spread onto MRS agar containing bromocresol purple, CaCO_3_, XLD, or LB agar and incubated at 42°C for 24 h–48 h to determine the total LAB, *Salmonella* spp., or total heterotrophic bacteria (THB) numbers, respectively. The number of bacteria was counted and expressed as log_10_ CFU per gram of wet cecum. The investigation of bacteria in the cecum was performed using six replicates of 15-, 30-, and 45-day-old broilers.

Genomic DNA from bacteria isolated from the cecum was extracted, and 16S rRNA was amplified using a pair of 16S rDNA primers (27F and 1492R). The 16S rRNA polymerase chain reaction product was sequenced. A phylogenetic tree was constructed using the neighbor-joining method based on the 16S rRNA sequences to compare the sequences of probiotics used in this experiment.

### Intestinal histology

Six 45-day-old broilers from each treatment and control groups were euthanized by cervical dislocation, and the intestine was collected immediately. Approximately 2-cm segments of the proximal part of the duodenum, jejunum, and ileum were cut, gently washed twice with 0.85% NaCl and opened longitudinally. The tissue samples were fixed in 10% (v/v) formalin for at least 48 h, dehydrated in a Shandon Citadel 1000 Tissue Processor through a series of ethanol concentrations from 70% to 100% (v/v) and soaked in 100% xylene. The dehydrated samples were embedded in paraffin using a Leica EG1150 H Heated Paraffin Embedding instrument (Leica Biosystems Inc., USA). The embedded samples were cut to a thickness of 5 μm with a Leica RM2235 rotary microtome. The tissue sections were deparaffinized and stained with hematoxylin and eosin using a standard procedure. Histological sections of the duodenum, jejunum, and ileum were observed and analyzed under a compound light microscope equipped with AxioVision Rel. 4.8 (ZEISS, Germany) to evaluate the villus height, cryptal depth, and villus height-to-cryptal depth ratio.

### Statistical analysis

The differences in mean values among groups were analyzed by one-way analysis of variance (ANOVA) followed by Fisher’s least significant difference (LSD) test for post-test comparisons (GraphPad, Prism version 9.111, San Diego, CA, USA, www.graphpad.com). The effects of interactions among the different parameters, dried probiotics, and administration programs were assessed by two-way ANOVA. The level of significance considered in all statistical analyses was set at p < 0.05.

## Results

### Effect of freeze- and spray-drying on the viable cell count and survival of probiotics

**Figure-1:**
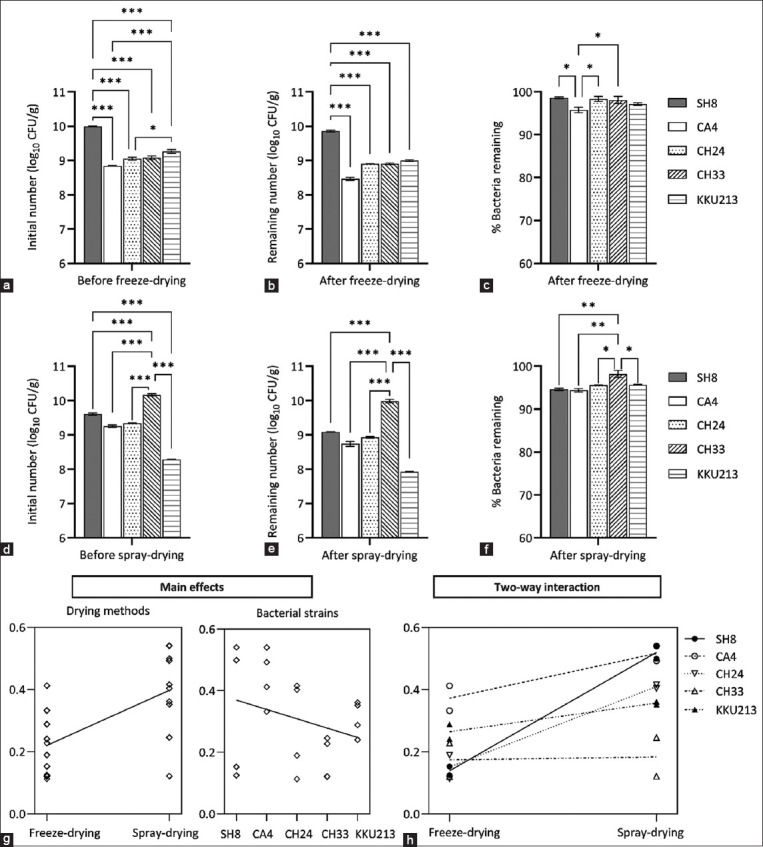
(a) Initial number before freeze-drying. (b) Remaining number and (c) Percent survival of probiotic strains after freeze-drying. (d) Initial number before spray-drying. (e) Remaingin number and (f) Percent survival of probiotic strains after spray-drying. (g) Plot of the main effects on reductions in the viable cell count obtained with different drying methods and bacterial strains. (h) Two-way interactions of the drying methods and bacterial strains. *, **, and *** indicate significance at p < 0.05, p < 0.01, and p < 0.001, respectively.

The five strains of probiotics used in this study showed significant differences in the initial cell counts before freeze- and spray-drying (Figure-1a and 1d). For freeze-drying, the four LAB strains (CA4, CH24, CH24, and SH8) were cultivated in MRS broth containing a mixture of prebiotics (rice bran, chitosan, and CMC) for 48 h, and one strain of a potential probiotic, *B. subtilis* KKU213, was cultivated in LB broth containing the mixture of prebiotic carriers for 18 h. The viable cell counts before freeze-drying showed significant (p < 0.05) differences among the probiotic strains (Figure-1a). The viable cell count of SH8 was 10.0 log CFU/g, which was significantly higher than that observed for the other LAB strains (9.1–8.8 CFU/g) and *B*. *subtilis* KKU213 (9.3 log CFU/g). For spray-drying, although the four LAB strains were cultivated in modified broth containing the prebiotic-carrier mixture under similar conditions for 48 h, the viable cell count of CH33 was significantly higher (10.17 log CFU/g) than those of the other LAB strains (9.6–9.3 log CFU/g). SH8, CA4, and CH24 showed no significant differences in the initial number of viable cells (Figure-1d). KKU213, which was cultivated in modified broth containing a similar prebiotic-carrier mixture for 18 h, presented a significantly lower viable cell count than the LAB strains cultivated in a similar medium for 48 h.

The percentage of viable bacteria of all strains remaining after freeze- and spray-drying ranged from 98.6% to 95.8% (Figure-1c) and 98.2% to 94.4% (Figure-1f), respectively. The numbers of viable cells were lower after freeze- and spray-drying and different from the initial cell count of each probiotic strain before the drying process. The viable cell counts in the freeze- and spray-dried probiotic powders after the drying processes used in this study were in the range of 9.9–8.9 log CFU/g (Figure-1b) and 10.0 to 7.9 log CFU/g (Figure-1e), respectively, which may be associated with the initial cell number of the probiotic before freeze- and spray-drying. The loss of viable cells in the immobilized dried probiotics was significantly affected by the drying method (F_1,10_ = 67.53, p < 0.0001), bacterial strain (F_4,10_ = 15.74, p = 0.0003) and the interaction between the drying method and bacterial strain (F_4,10_ = 9.18, p = 0.0023) (Figure-1g and h).

### Effect of the storage period and temperature on the survival of the immobilized probiotics

**Figure-2:**
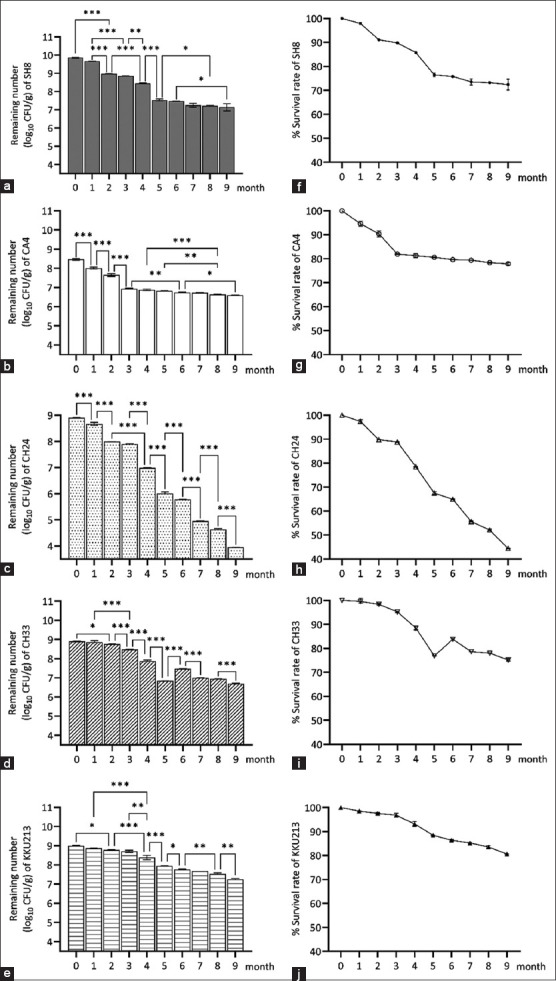
Effect of the storage time on (a-e) number of remaining cells and (f-j) percent survival rate of different freeze-dried probiotic strains. *, **, and *** indicate significance at p < 0.05, p < 0.01, and p < 0.001, respectively.

**Figure-3:**
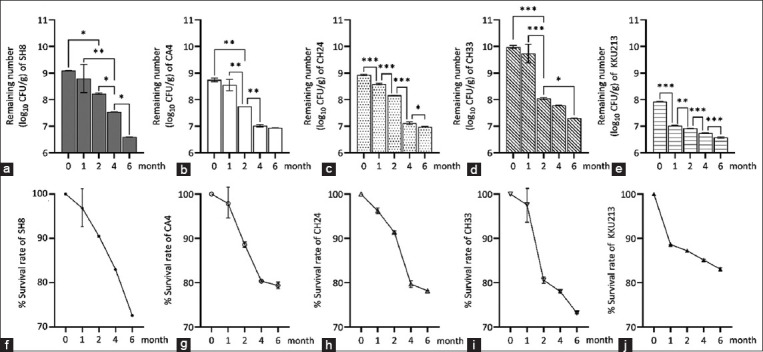
Effect of the storage time on the (a-e) number of remaining cells and (f-j) percent survival rate of different spray-dried probiotic strains. *, **, and *** indicate significance at p < 0.05, p < 0.01, and p < 0.001, respectively.

The viable cell counts and survival of the freeze- and spray-dried probiotics were markedly decreased after storage in a refrigerator at 4°C from 1 to 9 months (Figure-2) and in a desiccator at ambient air temperature from 1 to 6 months (Figure-3), respectively. The viable cell counts of freeze-dried SH8, CH33, and KKU213 stored at 4°C for 1 month were not significantly different from those at the beginning of the storage period. The reduction in viable cells obtained with spray-dried SH8, CA4, and CH33 stored in a desiccator at ambient air temperature was not significantly different from the reduction in viable cells from the beginning to one month after the storage. The percentage of surviving cells remaining in the freeze- and spray-dried probiotic powders of all strains after 1 month decreased from an initial cell survival of 100% to 99.6%–94.6% and 97.9%–88.6%, respectively.

After 6 months at 4°C, the viable cell count of the freeze-dried probiotics of SH8, CA4, CH24, CH33, and KKU213 showed average reductions of 2.4, 1.7, 3.1, 1.4, and 1.2 log, respectively (Figure-2a–e). The spray-dried probiotics stored for 6 months at ambient room temperature exhibited log reductions of 2.5, 1.8, 1.9, 2.7, and 1.3, respectively (Figure-3a–e). After 9 months, the numbers of surviving freeze-dried CH24 decreased by 4.9 log CFU/g, whereas a reduction of only 1.7 log CFU/g was observed for KKU213.

**Figure-4:**
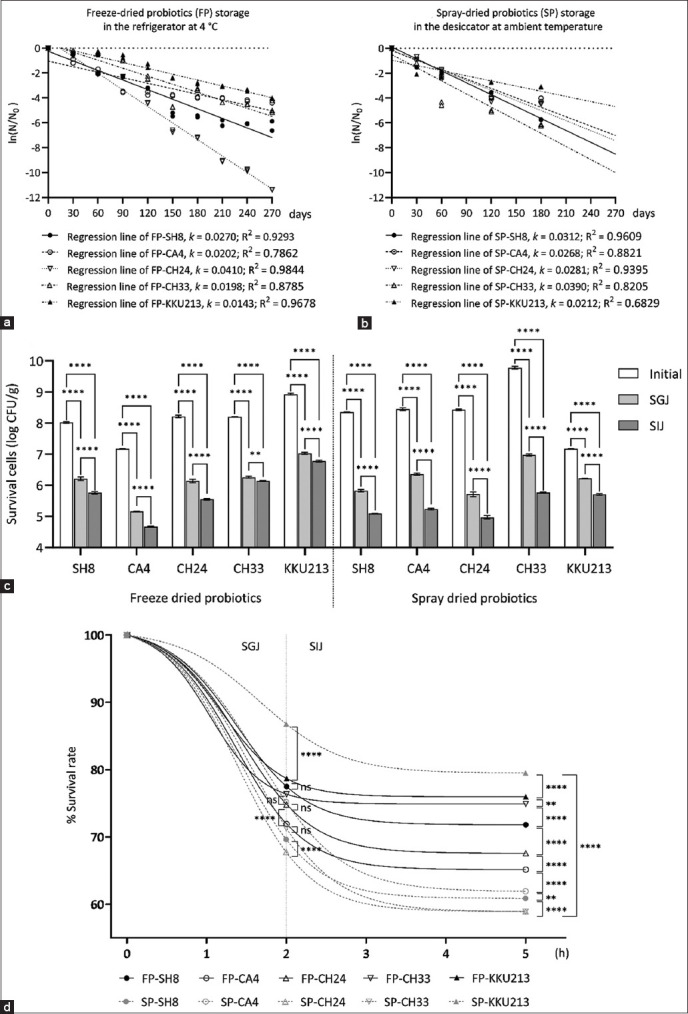
Regression lines representing the storage stability of (a) freeze-dried and (b) spray-dried probiotics after storage at 4°C for 9 months and at ambient temperature for 6 months. Number of (c) surviving cells and (d) survival rate of freeze-dried and spray-dried probiotics under *in vitro* SGJ (pH 2.0) and SIJ (0.5% bile salt) conditions. *, **, ***, and **** indicate significance at p < 0.05, p < 0.01, p < 0.001, and p < 0.0001, respectively. FP: freeze-dried; SP: spray-dried.

The freeze- and spray-dried KKU213 displayed low specific degradation rates (k values) of 0.0143 and 0.0212 during storage at 4°C and ambient air temperature, respectively, compared with the other freeze- and spray-dried LAB strains (Figure-4a and b). The loss of viable probiotic cells during prolonged storage under the two conditions was predicted from the regression lines, and the R^2^ values ranged from 0.98 to 0.68. As a result, the k values found for the freeze-dried powders of SH3, CA4, CH33, and KKU213 during storage at 4°C for 6 months were lower than those found for the spray-dried powders of each strain during storage at ambient air temperature in a desiccator for 9 months.

### Antimicrobial activity of immobilized probiotics

The freeze- and spray-dried SH8, CH24, and CH33 exhibited an inhibitory effect on the growth of all tested pathogens, including *S*. Typhimurium, *S*. Braenderup, and *S*. Enteritidis. Freeze- and spray-dried KKU213 exerted only a weak inhibitory effect on *S*. Typhimurium (Table-1).

**Table-1:** Antimicrobial activity of freeze- and spray-dried immobilized probiotics.

Strain	*Salmonella* Typhimurium ATCC13311		*S.* Braenderup H9812		*S.* Enteritidis T6	
		
	Freeze-dried	Spray-dried	Freeze-dried	Spray-dried	Freeze-dried	Spray-dried
CA4	++	++	-	-	++	++
CH24	+	+	+	+	+	+
CH33	+	+	+	+	+	+
SH8	++	++	+	++	++	++
KKU213	+	+	-	-	-	-

–=No inhibition, +=Colony diameter divided by the diameter of the colonies and zones in the range of 0 to 0.5 mm, ++=Colony diameter divided by the diameter of the colonies and zones in the range of 0.6–1.0 mm

### Survivability of immobilized probiotics under *in vitro* simulated gastrointestinal conditions

The immobilized probiotics were more resistant to SGJ and SIJ conditions than the free cells, and only free CH33 cells survived with a low survival rate after exposure to SGJ (data not shown). The SGJ condition at pH 2.0 led to a significant 1–2.8 log decrease in the survival rate of both freeze- and spray-dried probiotics (Figure-4c). The survival rate of freeze-dried KKU213 after 2 h of exposure to SGJ was 78.7 ± 0.6%, which was significantly higher than the rates observed for the freeze-dried strains CA4 (71.9%) and CH24 (to 74.8%). Among the spray-dried probiotics, KKU213 showed the highest survival rate (86.8 ± 0.0%) compared with all the other strains (67.8%–75.2%) after 2 h of exposure to SGJ conditions (Figure-4d).

All five bacterial strains were able to tolerate 0.5% bile salt conditions. A decline in the viable cell count ranging from 0.1 to 1.2 log was found for all probiotic strains as they were transferred from SGJ to SIJ conditions with 0.5% bile salt for 3 h (Figure-4c). Significant decreases in the viable bacteria of all bacterial strains to 4.7 and 6.8 log CFU/g and in the survival rates to 59.0 and 79.5% were found after exposure to *in vitro* SGJ for 2 h and SIJ for 3 h, respectively. The survivability of immobilized bacteria during exposure to the low pH and bile salt concentration used in this study depended on the bacterial species (F_4,10_ = 1577, p < 0.0001), drying method (F_1,10_ = 2763, p < 0.0001), and their interaction (F_4,10_ = 631.3, p < 0.0001).

### Effects of immobilized probiotics and administration program in male Cobb broilers

**Figure-5:**
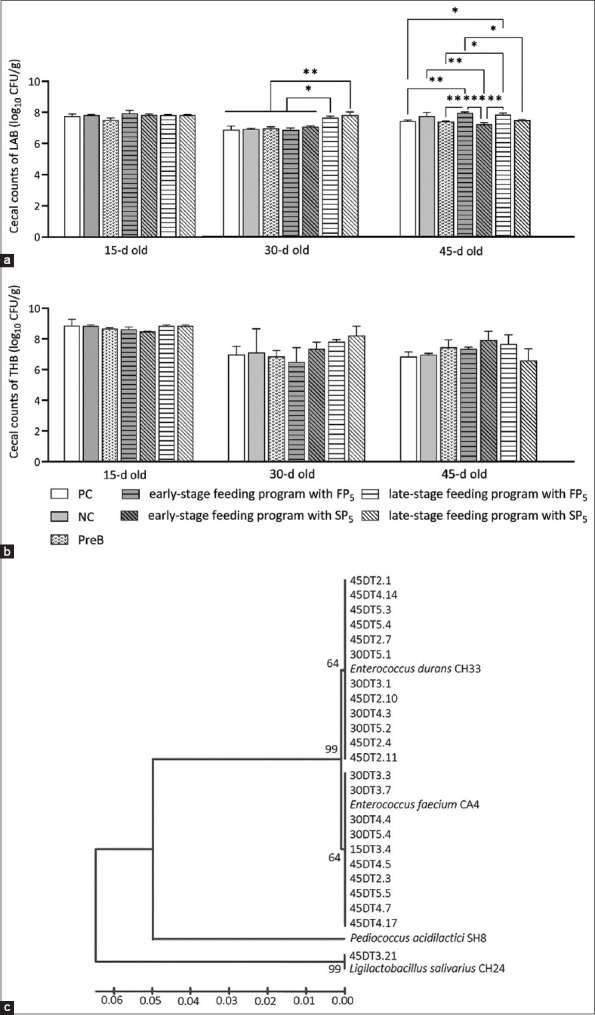
(a) Cecal counts of lactic acid bacteria (LAB) and (b) cecal counts of total heterotrophic bacteria in broilers and (c) phylogenetic tree of LAB isolated from the cecum of probiotic-supplemented broilers.

The BWG of the broilers at all weeks, except the 3^rd^ week, was significantly affected by the experimental diets or treatments (Table-2). The dietary inclusion of PreB, freeze- and spray-dried probiotics improved the final BWG after 6 weeks compared with that of the NC control group. At 30 days of age, a significant difference in the cecal LAB numbers was found between the probiotic-supplemented broiler group with the late-stage feeding program and all other groups. The cecal LAB numbers of the broilers at 45 days of age were increased in all the probiotic-supplemented broiler groups, except the SP_5_-fed group at the early-stage, compared with those of the PC group (Figure-5a). However, no difference in the cell number of THB was found among the groups (Figure-5b). Out of the 123 LAB strains isolated from the caeca of broilers at 15–45 days of age, 23 isolates showed a pattern of antibiotic susceptibility and antimicrobial activity similar to that found for our tested LAB probiotics. All 23 isolates were potentially identified as *E. faecium* CA4 (n = 10), *E. durans* CH33 (n = 12), and *L. salivarius* CH24 (n = 1), demonstrating the potential colonization of the tested LAB probiotics in the intestinal tract of the broilers (Figure-5c).

**Table-2:** Effect of probiotic-supplemented diets and feeding programs on the BWG, histomorphology of the small intestine and serological blood parameters of broilers.

Parameters	PC	NC	PreB	Early-stage		Late-stage		p-value
	
				FP_5_	SP_5_	FP_5_	SP_5_	
BWG (g)								
1^st^ week	138.2 ± 2.8^a^	118.7 ± 2.0^b^	123.7 ± 4.8^b^	126.0 ± 4.9^ab^	125.0 ± 3.7^ab^	118.7 ± 2.0^b^	118.7 ± 2.0^b^	**
2^nd^ week	310.5 ± 7.9^a^	280.5 ± 4.8^b^	335.0 ± 14.4^a^	323.7 ± 10.5^a^	308.5 ± 10.6^ab^	280.5 ± 4.8^b^	280.5 ± 4.8^b^	**
3^rd^ week	490.0 ± 13.0	484.7 ± 14.7	545.0 ± 26.3	499.2 ± 25.9	457.8 ± 22.1	490.9 ± 17.3	453.5 ± 23.7	ns
4^th^ week	731.3 ± 15.7^a^	676.7 ± 15.2^ab^	736.5 ± 25.6^a^	674.7 ± 20.5^ab^	587.5 ± 38.6^b^	685.5 ± 27.0^ab^	706.7 ± 42.2^ab^	**
5^th^ week	1008.7 ± 19.6^a^	786.3 ± 22.1^c^	866.1 ± 50.6^bc^	762.5 ± 28.1^c^	936.7 ± 40.3^ab^	839.4 ± 48.2^bc^	915.0 ± 38.6^ab^	**
6^th^ week	1197.3 ± 41.5^a^	947.5 ± 22.8^b^	991.7 ± 105.2^ab^	938.1 ± 52.4^b^	1101.7 ± 62.1^ab^	1074.4 ± 51.6^ab^	1071.9 ± 78.4^ab^	**
Total (g/6 weeks)	3638.2 ± 48.0^a^	3288.8 ± 45.2^c^	3597.5 ± 137.5^a^	3344.4 ± 62.5^bc^	3515.0 ± 109.8^ab^	3488.1 ± 87.7^ab^	3555.0 ± 74.9^ab^	**
Histomorphometry								
Duodenum								
Villus height (μm)	1307.0 ± 50.1^c^	1632.4 ± 111.8^ab^	1450.7 ± 109.9^bc^	1337.3 ± 25.0^c^	1719.7 ± 4.0^a^	1640.3 ± 57.5^ab^	1735.6 ± 41.1^a^	**
Cryptal depth (μm)	104.0 ± 0.5^b^	148.3 ± 7.4^a^	145.1 ± 7.4^a^	143.2 ± 9.3^a^	147.6 ± 3.6^a^	141.6 ± 4.4^a^	148.3 ± 5.0^a^	**
VH/CD	12.6 ± 0.5^a^	11.0 ± 0.5^bc^	9.9 ± 0.4^cd^	9.4 ± 0.7^d^	11.7 ± 0.26^ab^	11.6 ± 0.38^ab^	11.7 ± 0.22^ab^	**
Jejunum								
Villus height (μm)	1089.8 ± 69.0^a^	493.5 ± 4.2^c^	807.2 ± 46.0^bc^	793.8 ± 64.0^b^	790.6 ± 91.7^b^	961.2 ± 63.2^ab^	886.1 ± 45.9^b^	***
Cryptal depth (μm)	138.0 ± 6.6^a^	112.1 ± 3.9^bc^	90.9 ± 2.6^cd^	139.4 ± 6.4^a^	112.2 ± 4.3^b^	119.9 ± 9.3^ab^	125.7 ± 10.7^ab^	**
VH/CD	7.9 ± 0.1^ab^	4.4 ± 0.1^d^	8.9 ± 0.5^a^	5.8 ± 0.7^cd^	7.0 ± 0.6^bc^	8.1 ± 0.5^ab^	7.1 ± 0.5^bc^	***
Ileum								
Villus height (μm)	455.3 ± 29.0	398.9 ± 16.1	416.1 ± 8.7	416.1 ± 18.8	402.8 ± 4.4	451.9 ± 82.3	403.3 ± 33.0	ns
Cryptal depth (μm)	94.9 ± 4.4^bcd^	81.7 ± 3.5^d^	82.9 ± 1.2^cd^	103.3 ± 9.9^ab^	85.7 ± 3.5^cd^	118.7 ± 7.2^a^	98.4 ± 3.6^bc^	**
VH/CD	4.8 ± 0.4	4.9 ± 0.3	5.0 ± 0.1	4.1 ± 0.2	4.7 ± 0.2	3.8 ± 0.6	4.1 ± 0.3	ns
Serological blood parameters (units of mg/day)								
15 days old								
CHOL	152.0 ± 4.9^ab^	161.0 ± 4.0^a^	132.0 ± 13.1^b^	136.7 ± 6.7^b^	117.0 ± 15.2^c^	161.0 ± 4.0^a^	161.0 ± 4.0^a^	**
TG	36.0 ± 4.4^c^	70.3 ± 9.8^b^	103.0 ± 4.0^ab^	109.7 ± 19.9^a^	120.0 ± 15.5^a^	70.3 ± 9.8^b^	70.3 ± 9.8^b^	***
HDL	114.6 ± 4.7^a^	124.3 ± 4.3^a^	88.7 ± 8.4^b^	100.0 ± 7.8^b^	88.7 ± 14.9^b^	124.3 ± 4.3^a^	124.3 ± 4.3^a^	**
LDL	30.7 ± 2.5^a^	22.7 ± 3.0^ab^	22.7 ± 4.9^ab^	14.7 ± 5.5^bc^	4.2 ± 2.5^c^	22.7 ± 3.0^ab^	22.7 ± 3.0^ab^	**
Uric	6.6 ± 0.9^a^	2.0 ± 0.3^b^	6.2 ± 0.3^a^	4.1 ± 1.1^b^	5.7 ± 0.6^a^	2.0 ± 0.3^b^	2.0 ± 0.3^b^	***
30 days old								
CHOL	136.6 ± 7.9^a^	124.5 ± 3.2^ab^	103.3 ± 5.8^b^	114.9 ± 5.2^b^	104.9 ± 8.5^b^	117.1 ± 6.1^b^	109.0 ± 6.5^b^	*
TG	124.9 ± 4.5	108.0 ± 8.5	117.0 ± 3.0	118.1 ± 11.6	109.4 ± 9.3	92.3 ± 8.3	94.4 ± 16.8	ns
HDL	47.6 ± 3.2^a^	46.8 ± 1.4^a^	35.0 ± 3.0^b^	36.3 ± 3.1^b^	37.8 ± 3.1^b^	43.4 ± 2.3^ab^	38.7 ± 1.5^b^	**
LDL	64.1 ± 5.1	56.4 ± 2.4	44.8 ± 2.9	56.4 ± 3.1	46.6 ± 6.5	55.3 ± 5.6	51.6 ± 5.0	ns
Uric	4.7 ± 0.4	3.6 ± 0.3	4.0 ± 0.9	4.0 ± 0.4	4.0 ± 0.5	4.1 ± 0.3	5.0 ± 0.3	ns
45 days old								
CHOL	137.3 ± 6.1	136.3 ± 11.6	131.3 ± 9.4	133.3 ± 2.0	123.0 ± 14.2	124.7 ± 3.8	128.3 ± 7.3	ns
TG	92.3 ± 14.8	70.3 ± 15.4	85.0 ± 6.2	89.3 ± 19.5	76.3 ± 5.7	80.3 ± 0.9	117.0 ± 10.8	ns
HDL	78.0 ± 1.5	85.7 ± 3.4	82.7 ± 4.3	80.7 ± 2.2	76.3 ± 8.1	78.3 ± 2.4	72.7 ± 2.3	ns
LDL	40.7 ± 2.7	36.7 ± 6.4	31.3 ± 4.9	34.7 ± 4.4	31.3 ± 6.9	30.3 ± 1.6	32.0 ± 4.0	ns
Uric	4.1 ± 0.7	3.6 ± 0.3	3.8 ± 0.7	3.7 ± 0.3	4.8 ± 0.3	3.8 ± 0.4	5.2 ± 0.3	ns

Mean values in the same row with different letters are significantly different as determined by one-way ANOVA followed by Fisher’s LSD test. ns indicates a non-significant difference, * indicates a significant difference at p *<* 0.05,

** indicates a significant difference at p *<* 0.01, and

*** indicates a significant difference at p *<* 0.001. Body weight gain (g) were assessed every week for 6 weeks (42 days). Histomorphometry was measured for 45-day-old broilers. PC=Positive control administered the basal diet, NC=Negative control administered the basal diet without antibiotics, prebiotics, or probiotics, PreB=Basal diet with rice bran/chitosan/CMC, FP_5_=Basal diet with freeze-dried five-probiotic mixture, and SP_5_=Basal diet with spray-dried five-probiotic mixture. All serological blood parameters were assessed for 42-day-old broilers and presented in units of mg/day. VH/CD=Villous height-to-cryptal depth ratio, CHOL=Cholesterol, TG=Triglyceride, HDL=High-density lipoprotein, LDL=Low-density lipoprotein, BWG=Body weight gain

Significant (p < 0.01) increases in the duodenal villus height and duodenal cryptal depth were found in the probiotic-supplemented broilers treated using the two different feeding programs, except the FP_5_ group fed at the early-stage (Table-2). The serum CHOL and HDL concentrations were lower in the PreB and probiotic groups than in the PC and NC groups (Table-1). The concentration of TGs in the groups fed probiotics at the late-stage was lower than that observed in the other groups. The serum LDL and uric acid concentrations did not differ between the control and experimental groups. The results from a two-way ANOVA of the total BWG, small intestinal morphology, and serological parameters indicated the effects of the dried probiotics, administration programs, and their interaction (Table-3). However, no significant effect on the total BWG and serological parameters of broilers was derived from the two variables and their interaction. Nevertheless, the dried probiotics, administration programs, and their interaction had a significant effect on increases in the villus height and villus height-to-cryptal depth ratio in the duodenum.

**Table-3:** Results from a statistical analysis (p values) of the impact of dried probiotics (freeze- and spray-dried probiotics; FP_5_ and SP_5_), feeding programs (early-stage and late-stage programs), and their interaction on the growth performance, intestinal morphology, and serological parameters of 42-day- or 45-day-old broilers.

Parameters	df	Dried probiotics		Administration programs		Dried probiotics × administration programs	
		
		F-value	p-value	F-value	p-value	F-value	p-value
Total BWG	1	2.043	ns	1.223	ns	0.390	ns
Duodenum							
Villus height	1	40.47	***	18.04	**	14.62	**
Cryptal depth	1	0.867	ns	0.005	ns	0.037	ns
VH/CD	1	7.584	*	6.690	*	6.114	*
Jejunum							
Villus height	1	0.329	ns	3.715	ns	0.278	ns
Cryptal depth	1	1.763	ns	0.140	ns	4.188	ns
VH/CD	1	0.069	ns	4.126	ns	3.445	ns
Ileum							
Villus height	1	0.477	ns	0.165	ns	0.155	ns
Cryptal depth	1	8.161	*	4.460	ns	0.042	ns
VH/CD	1	1.578	ns	1.408	ns	0.182	ns
Blood parameters							
CHOL	1	0.163	ns	0.041	ns	0.719	ns
TG	1	1.060	ns	1.898	ns	4.669	ns
HDL	1	0.022	ns	0.442	ns	0.022	ns
LDL	1	0.033	ns	0.158	ns	0.293	ns
URIC	1	15.52	ns	0.644	ns	0.310	ns

df=degree of freedom; ns=non-significant difference,

* significant difference at p *<* 0.05,

** significant difference at p *<* 0.01, and

*** significant difference at p *<* 0.001

**Figure-6:**
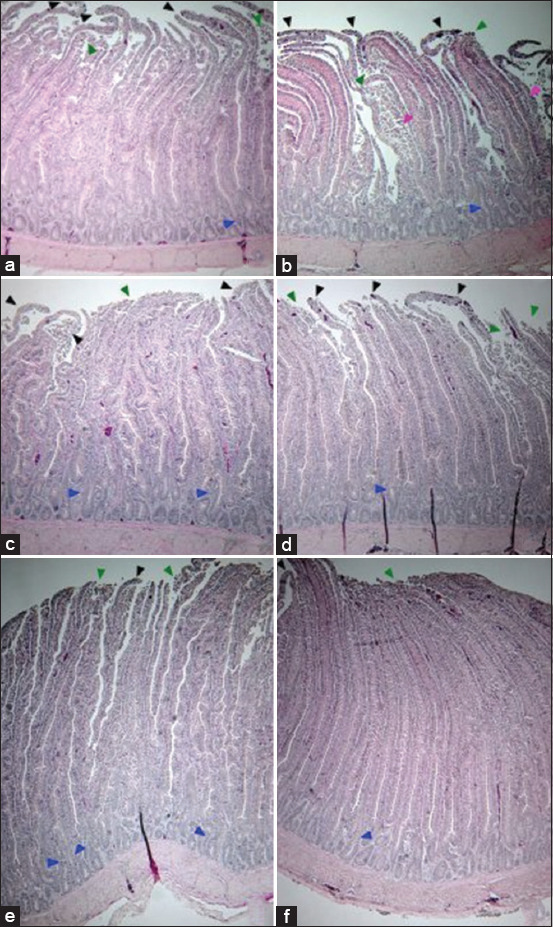
Histological micrographs of transverse sections from the duodenum of broilers in the control and experimental groups. (a) PC: Positive control fed the normal diet, (b) PreB: Diet with carrier, (c) early-stage feeding with FP_5_, (d) early-stage feeding with SP_5_, (e) late-stage feeding with FP_5_, and (f) late-stage feeding with SP_5_. Black arrowhead: Disruption of the apical villus, Green arrowhead: Disruption and erosion of the epithelium, Blue arrowhead: Fusion of the villus and crypt, Pink arrowhead: Swollen villus. FP_5_: Basal diet with five freeze-dried probiotic strains, SP_5_: Basal diet with five spray-dried probiotic strains.

In addition, micrographs of the duodenum of the different groups revealed the positive effect of probiotic-supplemented diets administered using the late-stage program on broiler gut health (Figure-6). The intestine of broilers in the PC group showed fusion in some villus tips with disruption and erosion of epithelial cells (Figure-6a). Loss of the cell membrane and the appearance of disrupted villus tips was mostly observed in the intestine of the PC and PreB groups and both the FP_5_ and SP_5_ groups fed based on the early-stage program (Figure-6b–f).

## Discussion

The differences in the initial cell number among probiotic strains may depend on the growth conditions at the cultivation step in the freeze- or spray-drying process [15]. The decrease in viable cell count depended on the drying method, bacterial strain and their interaction. This finding agrees with recent studies that revealed a difference in the loss of viability of probiotics due to thermal, cryo, mechanical, and oxidative damage induced by the drying process and the degree of tolerance of the probiotic species to drying [16, 17]. The freeze-drying process used in this study was more effective in terms of reducing the loss of viable cells. Similar to the results obtained by Iaconelli *et al*. [18], freeze-drying yielded the best maintenance of probiotic viability. The SH8 and CH24 strains are highly sensitive to heat stress during spray-drying. Celik and O’Sullivan [19] reported that most probiotic species are more sensitive to heat than cold stress, resulting in a higher degree of lethality following spray-drying compared with freeze-drying.

Rice bran, chitosan, and CMC, a polysaccharide that serves as a protectant and prebiotic-carrier, could protect bacterial cells from thermal stress during drying and enhance their survivability [10, 20, 21]. This study obtained a high percent survival rate (>90%) with freeze- and spray-dried probiotics. Polysaccharide carriers can create a multilayer coating on the bacterial cell surface that provides a more thermal protection by stabilizing the cell structure during drying [13]. The effectiveness of immobilization was affected by the bacterial strains, and the CH33 strain immobilized by a mixture of rice bran, chitosan, and CMC using both drying methods was more resistant to thermal stress than the other strains, as revealed by the survival rate or reduction in cell survival. This finding supports the concept that the protection efficacy of the carriers may depend on the specificity of the probiotic strain [22]. The protective effects of the same carriers varied among and depended on the different *Lactobacillus plantarum* strains [23].

The high temperature and humidity of the storage conditions can induce lethal injury that promotes loss of cell survival in freeze- and spray-dried probiotics throughout the storage period [13]. *Bacillus* *subtilis* KKU213 appears to be more tolerant during storage under cold and ambient air temperatures due to its ability to produce an endospore, which may lead to higher stability and survival rates during storage [24]. In addition, the low k values of freeze- and spray-dried *B*. *subtilis* KKU213 also support the notion that spore-forming *B*. *subtilis* exhibits higher storage stability.

In this study, the storage stability of the freeze-dried probiotics was higher than that of the spray-dried probiotics. These findings are similar to those reported by Kamil *et al*. [25], who found a lower k value for freeze-dried probiotics than spray-dried probiotics. The lower moisture content of freeze-dried probiotic powders may be associated with a higher survival rate, which could be beneficial for prolonged storage [26]. Dried probiotic powders prepared using the two drying methods may show differences in the degree of sublethal injury and the number of sublethal probiotics [18, 25], which could influence the stability of dried probiotics during storage. The *k* values of freeze-dried probiotics during storage at 25°C and 4°C are slightly different [25]. Therefore, it would be interesting to evaluate the stability of probiotics under storage conditions at room temperature. Even if the freeze-dried probiotics stored at 4°C exhibited the highest stability, storage at room temperature has a lower utility cost and is easier to apply.

The antimicrobial activity of probiotics is an important characteristic that benefits host health. Our results confirmed that the antimicrobial activity of the probiotics was specific to the pathogen species [27]. Our previous study by Khochamit *et al*. [28] demonstrated that the cell-free supernatant of a fresh culture of *B*. *subtilis* KKU213 exhibited antimicrobial activity against a variety of pathogens but had no inhibitory effect on *S*. Typhimurium, but dried cells of KKU213 could inhibit this pathogen [28]. These results suggest that the antagonistic activity of *B*. *subtilis* KKU213 against *S*. Typhimurium may involve cell-to-cell contact and interspecies quorum sensing [29]. Freeze- and spray-dried SH8 was the most effective at inhibiting all tested pathogens, but KKU213 showed less inhibitory activity and could inhibit only one tested pathogen, *S*. Typhimurium. The abilities of the LAB probiotic strains CA4, CH24, and CH33 after freeze and spray-drying against *S*. Typhimurium, *S*. Braenderup, and *S*. Enteritidis were similar to those of non-dried or fresh cultures of each strain, as revealed in our previous study by Buahom *et al*. [30]. The present findings suggest that the antimicrobial activity of most probiotic strains could be maintained after the drying process. This finding is in accordance with the results of previous studies demonstrating that the drying process has no effect on the ability of LAB probiotics to protect against pathogens [31]. However, freeze-dried SH8 showed lower inhibitory activity against *S*. Braenderup than the spray-dried strain. After drying, freeze-dried *Lactobacillus* *fermentum* exhibits lower antimicrobial activity against all tested pathogens than spray-dried *L. fermentum* [27]. Thus, this finding shows that the functionality of SH8 and other probiotics, including their antimicrobial activity, is differentially altered by different drying processes.

Freeze-drying is more effective for maintaining survival than spray-drying [16, 17]. However, the period of repairing freeze-dried cell damage after rehydration is longer than that for spray-dried cells [18]. Indeed, repair of the cell damage, including cell membrane damage and any oxidatively damaged biomolecules, must occur before engaging in reproduction, which leads to a longer period at the lag phase [32]. Metabolite production is correlated with the growth cycle of microorganisms [33], and the more extensive periods of repairing freeze-dried cells [18] can delay metabolic pathways during the exponential phase to produce primary and secondary metabolites, including bacteriocin. Because antimicrobial activity depends on the kinetics of growth [34], the longer lag phase found for damaged cells could influence antimicrobial activity at the specific period of growth. Moreover, bacteriocin expression is controlled by various environmental stress response systems, such as the RecA-dependent SOS response, after exposure to environmental stress [35]. Therefore, these results could support our findings regarding the difference in antimicrobial activity between freeze- and spray-dried probiotic SH8. A DNA repair pathway called the SOS response is inducible by two key regulators, LexA, a repressor, and RecA, an inducer. In response to DNA damage, RecA can bind to single-stranded DNA (ssDNA) and induce self-cleavage of LexA, causing LexA to be degraded at the damage site, activating certain SOS genes. An SOS response is recognized as one of the most important global stress responses. Recent studies have shown that the SOS response plays a key role in repairing DNA damage as well as other functions [36].

The pH of the proventriculus and gizzard of poultry is approximately 2.5–3.5, and the bile salt concentration in the poultry intestinal tract ranges from 0.175 to 0.008%. Resistance to strong acid and bile salts is an important characteristic of probiotic bacteria, which denotes their ability to grow and colonize the host intestinal tract. Polysaccharide carriers or prebiotics, such as sugar, maize, CMC, chitosan, and rich bran, can protect bacterial cells from extremely low pH and high bile salt concentrations [37]. The ability of all immobilized bacteria to survive under the strong acid condition used in this experiment (pH 2.0) indicates their potential for colonization after transport from the stomach to the intestinal tract.

The difference in survivability among all freeze- and spray-dried strains may depend on their susceptibility to acid stress or their ability to respond to external environmental stress [38]. In addition, the resistance characteristics of the polysaccharide carriers or prebiotics used in this study (rice brane/chitosan/CMC) to the acidity of gastric juice and hydrolysis enzymes yielded a protective effect on the bacterial cells entrapped by the cross-linking or aggregation of rice brane/chitosan/CMC [39]. The negatively charged groups in the polysaccharide chains, such as CMC containing –CH_2_COO^-^, may prevent hydrogen ions from encountering the bacterial cells inside the cross-linked particles of the immobilization carriers [40] and buffer the bacterial cell from harmful environments. Similar to the results of a previous study by Gul and Atalar [16], the reduction in the survival of immobilized cells in the presence of bile salts was affected by the immobilization carriers and drying methods. Furthermore, the resistance of *B*. *subtilis* KKU213 and four LAB strains to bile salt might be explained by the bile salt extrusion mechanism and the hydrolysis of bile salts by bile salt hydrolase (BSH) [38].

All other bacterial strains immobilized using freeze- and spray-drying and exposed to pH 2 and 0.5% bile salts exhibited a survival rate higher than 50%, which indicated that they are good probiotic candidates with the potential to colonize the poultry intestine. Freeze-drying appeared to induce a better beneficial effect on almost all probiotic strains under *in vitro* SGJ and SIJ conditions, as revealed by higher survivability. The highest survivability was observed with spray-dried *B*. *subtilis* KKU213. The drying techniques used for bacterial immobilization may affect the behavior of carrier materials to produce microstructure through different mechanisms [41]. It is hypothesized that the capacity of carrier material to protect and deliver bacterial cells may vary depending on the microstructure of cross-linking between bacterial cells and carriers obtained using the drying method and their specific interaction.

The administration of both FP_5_ (basal diet with freeze-dried five-probiotic mixture) and SP_5_ (basal diet with spray-dried five-probiotic mixture) effectively enhanced the growth performance of broilers. This result concurs with the findings reported by Elbaz and El-Sheikh [42] and Bonos *et al*. [43], who found that diets supplemented with antimicrobials, whether antibiotics or probiotics, promote improvements in the performance of broilers, such as improvements in the body weight and feed conversion ratio. As observed in this study, probiotics that colonize the digestive tract may enhance the intestinal activity of the host by increasing the activity of digestive enzymes, the availability of nutrients, and the nutrient absorption ability [28, 42].

The reductions in the CHOL and TG levels observed in the current study were similar to those reported by Alaqil *et al*. [44], who found reduced CHOL and TG levels in poultry-fed diets with probiotic supplementation. The inclusion of LAB and *B*. *subtilis* in poultry diets reduces the serum CHOL levels by assimilating CHOL into the bacterial cell membrane, converting CHOL into coprostanol, and producing short-chain fatty acids (SCFAs) by the bacteria [45, 46], and the increase in SCFA production can inhibit the synthesis of CHOL in the host liver. In addition, the activity of BSH by probiotics leads to a reduction in the host CHOL level because CHOL is considered a precursor for bile salt synthesis that is needed for the maintenance of bile salt homeostasis [45]. The diet containing prebiotics (PreB) reduced the serum CHOL level in broilers, and this effect may be associated with an increase in the SCFA levels in the digestive tract [46]. In addition, the absorption of CHOL and bile acid hinders CHOL absorption into circulation and increases the content of CHOL and bile salt in feces [47]. Moreover, administering prebiotics or probiotics can alter lipid metabolism or reduce the digestion and absorption of ingested lipids in the host intestinal tract, which may be reflected by the observed reductions in the serum CHOL and TG levels in broilers [48]. As observed in the present study, serum uric acid level was not affected by the probiotic-supplemented diet administered using two different feeding programs [49]. These findings indicate that adding a mixture of five-probiotic strains could not improve the protein metabolism and functionality of the kidney. The results of an earlier study demonstrated that the protein metabolism represented by the uric acid levels significantly differed among the treatment groups fed diets supplemented with different probiotic mixtures and concentrations [49]. Therefore, the inconsistent effects of the probiotics on protein metabolism might be associated with the concentrations of the probiotics, the species and strains in the probiotic mixture, the administration methods, and/or the probiotic-host interactions. However, the revealed reductions in the CHOL level of broilers fed probiotics using the late-stage program reflected an improvement in animal health. Moreover, the low level of CHOL in the blood serum of broilers may be related to the meat quality and may contribute to healthy lean chicken meat.

Probiotics can alter intestinal morphology or intestinal epithelium thickness based on nutrient absorption and feed efficiency [43, 50]. The increased duodenal villus height of the groups administered probiotics (Table-2) may indicate an improvement in the nutrient absorption and growth performance of the probiotic-supplemented broilers. Park *et al*. [50] reported results consistent with our finding that the administration of *Lactobacillus* *sakei* Probio-65 maintains healthy gut conditions with more intact villi in the small intestine of broilers.

## Conclusion

Immobilization with rice bran/chitosan/CMC using freeze- or spray-drying processes could be useful for the preservation of probiotics for prolonged storage and protecting them from harmful environments, such as strong acid conditions and high bile salt concentrations. After immobilization using different drying processes, antimicrobial activity was still observed in all probiotic strains. The immobilized probiotics can be successfully delivered to the small intestine of broilers. The *E. durans* CH33 strain was isolated from the cecum of probiotic-supplemented broilers, which suggests its potential colonization. The administration of a mixture of probiotics using the late-stage feeding program (3^rd^ week) enhanced the growth performance, reduced the CHOL level, increased the duodenal villus height, and maintained the intestinal morphology. Based on the overall results, the cost-effective probiotics prepared by spray-drying were able to supplement the basal diet and feed administered using the late-stage program and serve as growth promoters to improve broiler health. Our findings provide an interesting alternative to the administration of commonly used antibiotics in broiler production.

## Data availability

The supplementary data are available from the corresponding author on a reasonable request.

## Authors’ Contributions

JB: Sample collection, methodology, investigation, data analysis, and writing of the original manuscript. SS: Conceptualization, methodology, resources, and validation. PS: Conceptualization and validation. TS: Validation, review, and editing of the manuscript. WS: Conceptualization, supervision, funding acquisition, project administration, review, and editing of the manuscript. All authors have read, reviewed, and approved the final manuscript.
